# RAVE and Rabconnectin-3 Complexes as Signal Dependent Regulators of Organelle Acidification

**DOI:** 10.3389/fcell.2021.698190

**Published:** 2021-06-24

**Authors:** Michael C. Jaskolka, Samuel R. Winkley, Patricia M. Kane

**Affiliations:** Department of Biochemistry and Molecular Biology, SUNY Upstate Medical University, Syracuse, NY, United States

**Keywords:** organelle acidification, Rabconnectin-3, vacuole, endosome and lysosome, V-ATPase, RAVE = regulator of H^+^-ATPase of vacuoles and endosomes, DMXL2, WDR7

## Abstract

The yeast RAVE (Regulator of H^+^-ATPase of Vacuolar and Endosomal membranes) complex and Rabconnectin-3 complexes of higher eukaryotes regulate acidification of organelles such as lysosomes and endosomes by catalyzing V-ATPase assembly. V-ATPases are highly conserved proton pumps consisting of a peripheral V_1_ subcomplex that contains the sites of ATP hydrolysis, attached to an integral membrane V_*o*_ subcomplex that forms the transmembrane proton pore. Reversible disassembly of the V-ATPase is a conserved regulatory mechanism that occurs in response to multiple signals, serving to tune ATPase activity and compartment acidification to changing extracellular conditions. Signals such as glucose deprivation can induce release of V_1_ from V_o_, which inhibits both ATPase activity and proton transport. Reassembly of V_1_ with V_o_ restores ATP-driven proton transport, but requires assistance of the RAVE or Rabconnectin-3 complexes. Glucose deprivation triggers V-ATPase disassembly in yeast and is accompanied by binding of RAVE to V_1_ subcomplexes. Upon glucose readdition, RAVE catalyzes both recruitment of V_1_ to the vacuolar membrane and its reassembly with V_o_. The RAVE complex can be recruited to the vacuolar membrane by glucose in the absence of V_1_ subunits, indicating that the interaction between RAVE and the V_o_ membrane domain is glucose-sensitive. Yeast RAVE complexes also distinguish between organelle-specific isoforms of the V_o_ a-subunit and thus regulate distinct V-ATPase subpopulations. Rabconnectin-3 complexes in higher eukaryotes appear to be functionally equivalent to yeast RAVE. Originally isolated as a two-subunit complex from rat brain, the Rabconnectin-3 complex has regions of homology with yeast RAVE and was shown to interact with V-ATPase subunits and promote endosomal acidification. Current understanding of the structure and function of RAVE and Rabconnectin-3 complexes, their interactions with the V-ATPase, their role in signal-dependent modulation of organelle acidification, and their impact on downstream pathways will be discussed.

## V-ATPases and Their Regulation by Reversible Disassembly

The endocytic pathway consists of a number of organelles that become progressively more acidic as they mature, with the lysosome as the terminal and most acidic compartment in the pathway (Huotari and Helenius, 2011). Organelle acidification is tightly associated with protein sorting and organelle function (Casey et al., 2010). Ligands dissociate from their receptors at a distinct pH range that helps to dictate their ultimate targeting (Sorkin and Von Zastrow, 2002), and the luminal pH of endosomes can drive association of trafficking factors themselves (Hurtado-Lorenzo et al., 2006). Hydrolytic enzymes are activated at the low pH of the lysosome and late endosomes, and H^+^-driven antiporters exert a more general control over the ionic environment in organelles (Li and Kane, 2009; Casey et al., 2010). Endosome acidification is critical for developmental signaling via the Notch and Wnt pathways (Niehrs and Boutros, 2010; Sun-Wada and Wada, 2015) but is also exploited by viruses to support release of their genetic material into the cytosol (Cossart and Helenius, 2014). Importantly, all acidic compartments of the endocytic pathway, as well as a several other organelles such as the late Golgi and regulated secretory granules, are acidified by V-ATPases, dedicated proton pumps that couple hydrolysis of cytosolic ATP to proton transport from the cytosol to the organelle lumen. Thus, V-ATPases are central players in organelle identity and function, signaling, and protein trafficking in the endocytic pathway.

**FIGURE 1:**
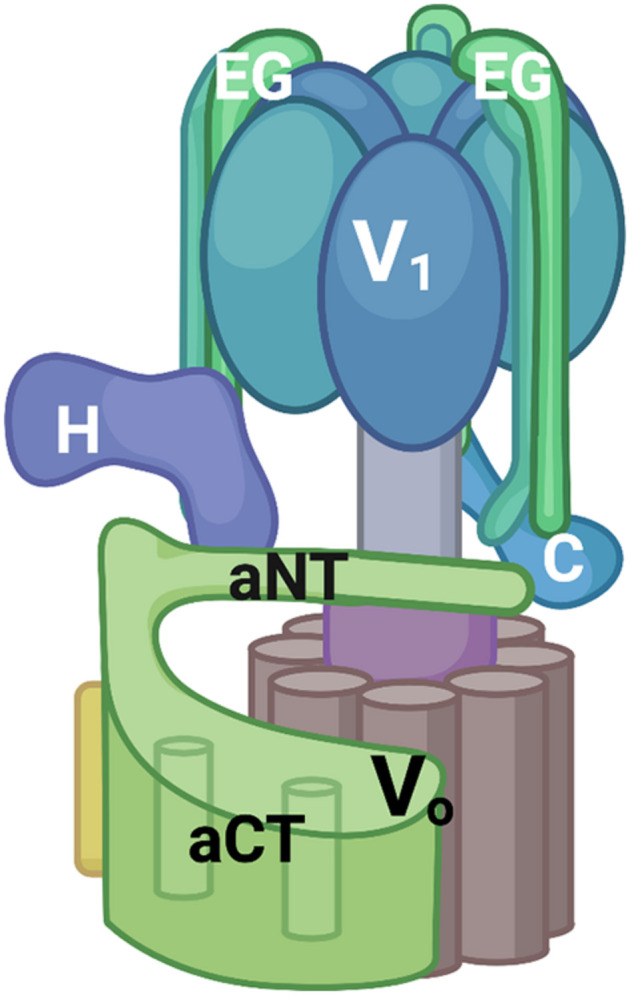
V-ATPase structural model. V-ATPases consist of a peripheral V_1_ complex and an integral membrane V_o_ complex, both containing multiple subunits. The V_1_ complex contains a hexamer of catalytic and regulatory subunits (in blue, not individually labeled) as well as three peripheral stalks each consisting of an EG heterodimer. Two of these EG heterodimers are labeled and the top of the third is visible at the back of the complex. The C and H subunits help to attach the V_1_ subcomplex to V_o_ serve important regulatory roles. The V_o_ subcomplex includes a ring of proteolipid subunits (brown) and the a-subunit. The a-subunit is a two-domain protein with a cytoplasmic domain (aNT) and an integral membrane domain (aCT). All figures were constructed using Biorender.com.

Both the subunit sequences and overall structure of eukaryotic V-ATPases are remarkably conserved. V-ATPases consist of a peripheral membrane complex, V_1_, and an integral membrane complex, V_o_. V_1_ contains three catalytic sites for ATP hydrolysis and V_o_ contains the proton pore. Several recent cryo-EM structures have supported the fundamental structural similarity between fungal and mammalian V-ATPases (Zhao et al., 2015; Abbas et al., 2020; Wang L. et al., 2020; Wang R. et al., 2020). As shown in Figure 1, the V_1_ complex features a hexamer of alternating catalytic and regulatory subunits. In the center of the V_1_ complex there is a central stalk that transmits conformational changes driven by ATP hydrolysis into rotation of a ring of proteolipid subunits in the V_o_ complex, thus driving proton transport. In addition, eukaryotic V-ATPases have three peripheral stalks containing the V_1_ E and G subunits; these peripheral stalks have distinct interactions with V_1_ “bridging” subunits C and H and the V_o_ a-subunit. The presence of multiple isoforms of several V-ATPase subunits creates organelle- and tissue-specific V-ATPases with distinct catalytic and regulatory properties (Marshansky and Futai, 2008). The V_o_ a-subunit, in particular, is frequently present as multiple isoforms (Toei et al., 2010). This subunit occupies a critical position at the interface of the V_1_ and V_o_ subcomplexes. It also binds multiple regulatory factors including glycolytic enzymes (Su et al., 2003; Lu et al., 2004) and phosphoinositide phospholipids (Li et al., 2014; Banerjee and Kane, 2017).

**FIGURE 2:**
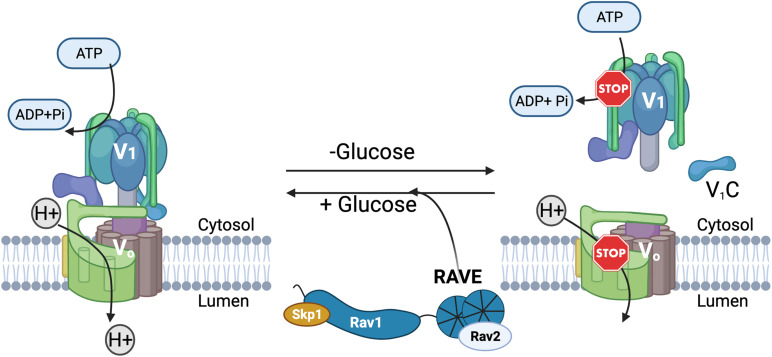
Reversible disassembly of the yeast V-ATPase. Active, assembled V-ATPases **(left)** couple hydrolysis of ATP to H^+^ transport across membranes. In yeast, glucose deprivation triggers disassembly and inactivation of some of the V-ATPase complexes. The V_1_ complex and subunit C are separately released from the membrane-bound V_o_ subcomplex into the cytosol, and both ATP hydrolysis and H^+^-transport are inhibited **(right)**. Upon glucose readdition, the RAVE complex helps to catalyze V-ATPase reassembly and reactivation.

In addition to similar structures, V-ATPases share a number of common regulatory mechanisms. Reversible disassembly is one of the best-studied mechanisms (Oot et al., 2017). Reversible disassembly was first discovered in the tobacco hornworm, *Manduca sexta* (Sumner et al., 1995), and the yeast *S. cerevisiae* (Kane, 1995) and is diagrammed in Figure 2. In both yeast and *M. sexta*, glucose deprivation triggers a rapid release of a large proportion of the peripheral V_1_ complexes from the membrane (Kane, 1995; Sumner et al., 1995). In addition, V_1_ subunit C detaches from both V_1_ and V_o_ (Kane, 1995; Voss et al., 2007). Remarkably, restoration of glucose results in equally rapid reassembly of the V_1_ complex and subunit C with the V_o_ complex at the membrane to reassemble the active V-ATPase holoenzyme (Kane, 1995; Sumner et al., 1995). V-ATPase disassembly in response to glucose deprivation appears to be an energy conservation mechanism. ATPase activity is inhibited in the cytosolic V_1_ subcomplexes and the V_o_ subcomplexes are closed to proton transport (Graf et al., 1996; Parra et al., 2000; Couoh-Cardel et al., 2015). In yeast, vacuolar pH is elevated after glucose deprivation but drops to a lower steady state pH within 90 sec of glucose readdition (Tarsio et al., 2011), indicating that reversible disassembly adjusts organelle acidification in response to extracellular conditions.

Since the initial characterization of reversible disassembly, it has become clear that this is a general mechanism for regulating V-ATPase activity that operates many different settings and in response to many different signals (Collins and Forgac, 2020). Early experiments on plasma membrane V-ATPases in kidney epithelial cells, where V-ATPases drive proton export, identified a similar glucose response to that observed in yeast and insects, with higher levels of V-ATPase activity and assembly at elevated extracellular glucose (Nakamura, 2004; Sautin et al., 2005). In contrast, more recent experiments on lysosomal V-ATPases in HEK293T and LLCPK cells found increased assembly and activity of V-ATPases under conditions of glucose deprivation (McGuire and Forgac, 2018). In this setting, increased lysosomal V-ATPase assembly and acidification may be a response to starvation that supports autophagic flux of nutrients. Extracellular glucose is not the only condition driving reversible disassembly of V-ATPases. In HEK293T cells, amino acid deprivation promotes increased assembly of lysosomal V-ATPases, likely as a means to promote protein degradation and amino acid recycling (Stransky and Forgac, 2015). In cardiomyocytes, high palmitate levels result in V-ATPase disassembly and endosome alkalinization, resulting in relocation of a lipid transporter from endosomes to the plasma membrane (Liu et al., 2017; Wang S. et al., 2020). In neurons, synaptic vesicles contain V-ATPases that drive neurotransmitter uptake, and reversible disassembly of these V-ATPases occurs as part of the synaptic vesicle cycle. Synaptic vesicle V-ATPases disassemble in preparation for fusion with the plasma membrane and reassemble to drive reloading of synaptic vesicles with neurotransmitter after endocytosis (Bodzeta et al., 2017). Importantly, in each of these settings, regulating the level of V-ATPase assembly serves to tune acidification of the compartment to specific cellular needs.

Given the diverse settings for reversible disassembly, it is not surprising that the signals triggering this process are also diverse [reviewed in Collins and Forgac (2020)] and in most cases incompletely understood. A number of signaling pathways are implicated in specific reversible disassembly events (Collins and Forgac, 2020). However, the molecular basis of their effects on V-ATPase assembly state is generally not well-understood. In insect cells, phosphorylation of a specific V-ATPase subunit, subunit C, been directly associated with reassembly, but this may be the only case where direct modification of a V-ATPase subunit correlates with assembly state (Voss et al., 2007, 2009).

On a structural level, reversible disassembly requires breaking and reforming many subunit-subunit interactions at the V_1_-V_o_ interface (Oot et al., 2017). The molecular order of events has been addressed in some detail but is still not completely clear. It has been proposed that disrupting the interaction of subunit C with aNT (the cytosolic N-terminal domain of the a-subunit) and one of the EG stalks could trigger a cascade of conformational changes that culminates in release of free subunit C and the V_1_ subcomplex into the cytosol during disassembly (Oot et al., 2017). Only catalytically active V-ATPases disassemble in response to glucose deprivation (Parra and Kane, 1998), suggesting that the V-ATPase holoenzyme is susceptible to dissociation only at certain points in the catalytic cycle. Three distinct conformations of the intact V-ATPase holoenzyme, which are believed to correspond to specific rotational positions, are observed by cryo-EM (Zhao et al., 2015). Significantly, the disassembled V_1_ and V_o_ complexes are each arrested at a single position, and the positions of the disassembled V_1_ and V_o_ match different conformations of the assembled enzyme (Mazhab-Jafari et al., 2016; Oot et al., 2016; Roh et al., 2018). This suggests that there is a conformational mismatch that must be overcome during reassembly.

In addition, significant conformational changes occur to silence the activities of the disassembled V_1_ and V_o_ complexes. In the V_1_ complex, the C-terminal domain of subunit H rotates 150° into an inhibitory conformation that traps ADP at one catalytic site and prevents ATP hydrolysis (Oot et al., 2016). In the V_o_ complex, aNT collapses toward the central stalk (Couoh-Cardel et al., 2015; Stam and Wilkens, 2016), and the c-ring assumes a distinct position relative to the membrane domain of the a-subunit (Mazhab-Jafari et al., 2016; Roh et al., 2020). Thus, reassembly of the V-ATPase holoenzyme requires a relief of the inhibitory conformations in both V_1_ and V_o_, reformation of the many subunit-subunit interactions that were broken, and a realignment of the rotational states of V_1_ and V_o_ complexes. Given the complexity of this process, it is not surprising that disassembled V_1_ and V_o_ subcomplexes do not readily reassemble *in vitro*. *In vitro* reassembly with restoration of V-ATPase activity has been seen under harsh conditions that partially dissociate the V_1_ complex (Parra and Kane, 1996), and thus do not mimic the physiological process. More recently, *in vitro* reassembly was achieved by using V_1_ complexes containing a mutant form of subunit H that cannot assume the inhibitory conformation (Sharma et al., 2019). Taken together, these data suggest that other cellular factors may be needed for reversible disassembly and point toward the energetic hurdles that these factors may help overcome.

## The Yeast RAVE Complex

### Discovery and Initial Characterization of the RAVE Complex

The yeast RAVE complex is composed of Rav1, Rav2, and Skp1, with Rav1 as the central component. It was discovered in a search for interacting partners for Skp1, an adaptor protein of SCF (Skp1-cullin-F-box) ubiquitin ligases (Seol et al., 2001). Two previously uncharacterized proteins, unrelated to SCF complexes and ultimately named Rav1 and Rav2, were identified from among many proteins co-isolated with yeast Skp1. Under less stringent isolation conditions, additional proteins that coprecipitated with the RAVE complex were determined to be V_1_ subunits (Seol et al., 2001).

In higher eukaryotes, complete loss of V-ATPase activity is lethal (Davies et al., 1996; Sun-Wada et al., 2000; Madsen and Gitlin, 2008). However, in yeast, loss of V-ATPase activity results in a Vma^–^ growth phenotype, characterized by slow growth under all conditions, optimal growth at pH 5, and failure to grow at pH 7.5 or in the presence of elevated calcium concentrations (Nelson and Nelson, 1990). Deletion of *RAV1* and *RAV2* resulted in a Vma^–^ phenotype, but at high temperature (37°C)(Seol et al., 2001). The source of the temperature sensitivity is still not clear. Importantly, *rav1*Δ and *rav2*Δ mutants proved to have a V-ATPase assembly defect (Seol et al., 2001; Smardon et al., 2002). After glucose deprivation and readdition, there was slow and incomplete reassociation of V_1_ subunits with the vacuolar membrane in a *rav1*Δ strain (Seol et al., 2001). Vacuolar vesicles isolated from *rav1*Δ and *rav2*Δ mutants have very low V-ATPase activity and reduced levels of V_1_ subunits compared to wild-type cells (Smardon et al., 2002). Consistent with this, *rav1*Δ mutant cells briefly deprived of glucose cannot acidify the vacuole upon glucose addition and instead show an increase in vacuolar pH upon glucose readdition, similar to that seen in V-ATPase mutants (Smardon et al., 2014). Taken together, these data indicate that the RAVE complex not only interacts with V-ATPase subunits but is also important for V-ATPase assembly and acidification of the vacuole.

**FIGURE 3:**
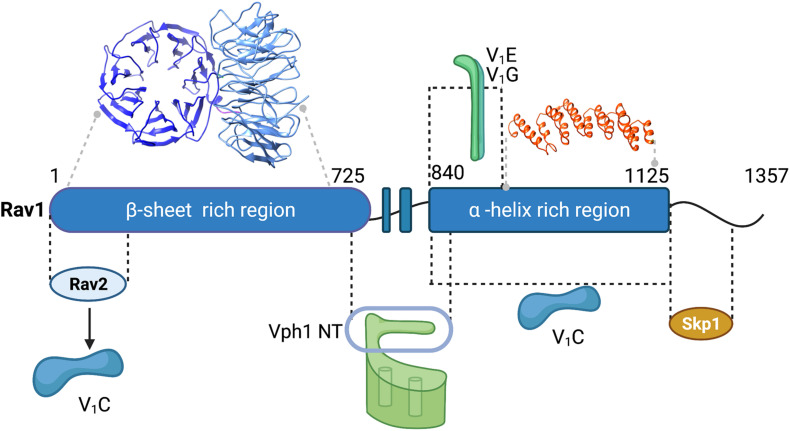
Map of interactions between yeast RAVE subunits and the V-ATPase and structural modeling of Rav1. Regions of interaction between subunits of the RAVE complex and between RAVE and V-ATPase subunits (labeled in Figure 1) are shown (Smardon et al., 2015). Structural models for two regions of Rav1 are also shown. Amino acids 2-672 of Rav1 were modeled on the double propeller of Apaf-1 (apoptotic protease activating factor-1;PDB 5JUY) by the Phyre2 server (Kelley et al., 2015). There is 14% amino acid sequence identity in this region, which was modeled with 100% confidence. In the view shown, the first β-propeller is viewed from the top, while the second β-propeller is viewed from the side. Amino acids 937–1113 of Rav1 were modeled on the α-solenoid region of chain A of gemin-5 (PDB 6RNS). There is 18% amino acid sequence identity with this region of Rav1, and it was modeled to the single highest scoring template with a confidence estimate of 98.7%. Black lines indicate regions exhibiting protein-protein interactions and gray lines indicate regions corresponding to the homology models.

There is still no high-resolution structure of RAVE or any related complex. Initial affinity purifications from wild-type and mutant cells indicated that Rav2 and Skp1 both interact with Rav1 but not with each other (Seol et al., 2001). Rav1 is also the largest subunit, with a predicted molecular mass of 154 kDa. Sequence comparisons and structural modeling have provided some insights into Rav1 structure. Secondary structure predictions indicated that the first 725 amino acids of Rav1 have a strong propensity to form β-sheet, amino acids 835–1195 are likely to have a high proportion of α-helices, and the C-terminal ∼150 amino acids are likely to be highly disordered. The β-sheet region of Rav1 can be modeled as a double β-propeller with high confidence and amino acids 937–1113 were modeled as an α-solenoid (Figure 3). Consistent with the initial pull-downs (Seol et al., 2001), Skp1 and Rav2 bind at opposite ends of Rav1 (Smardon et al., 2015). C-terminal deletions of Rav1 compromise Skp1 binding, and Rav2 binds to the N-terminal end of Rav1 *in vitro*. *RAV1* overexpression is lethal because excess Rav1 can bind Skp1 and prevent its binding to other essential complexes (Brace et al., 2006). The role of Skp1 in the RAVE complex was probed by selecting for a *skp1* mutation that could suppress the lethality of *RAV1* overexpression. The *skp1 S3R* mutation appeared to allow Skp1 participation in essential SCF ubiquitin ligase complexes, while preventing Skp1 binding to RAVE. Interestingly, this mutant had a relatively mild effect on RAVE function (Brace et al., 2006). Although mutants lacking Rav2 exhibit phenotypes similar to *rav1*Δ mutants, the function of Rav2 in the RAVE complex is still not clear.

### Mapping Interactions of RAVE With the V-ATPase

Interactions between the yeast RAVE and the V-ATPase were mapped through a combination of several approaches, and the mapped interactions are shown in Figure 3. The RAVE complex co-immunoprecipitates with cytosolic V_1_ complexes (Seol et al., 2001; Smardon et al., 2002). Deletion of the peripheral stalk subunits E and G disrupts this interaction (Smardon et al., 2002). The importance of the E and G subunits for the RAVE-V_1_ interaction is further supported by two-hybrid interactions between Rav1 and subunits E and G (Smardon and Kane, 2007). These data indicate that RAVE interacts with V_1_ through one or more of the three peripheral stalks. C-terminal truncations of Rav1 and two-hybrid assays with fragments of Rav1 indicate that amino acids 840–940 of Rav1 are important for interactions with subunits E and G (Smardon et al., 2015). Both Rav1 and Rav2 exhibit strong two-hybrid interactions with V_1_ subunit C, indicating that there may be two potential interaction sites for this subunit (Smardon and Kane, 2007). An expressed fragment containing amino acids 840–1125 of Rav1 also pulls down subunit C *in vitro*, suggesting that the binding sites for V_1_ subunits E, G, and C are likely to be in close proximity on Rav1 (Smardon et al., 2015).

The RAVE complex also interacts with the V_o_ a-subunit, and importantly, this interaction appears to be isoform-specific. *S. cerevisiae* has a single set of subunit isoforms for the V_o_ a-subunit, Vph1 and Stv1 (Manolson et al., 1994). Vph1 is expressed at higher levels and is predominantly found in the vacuole, while Stv1 resides in the Golgi at steady state. The cytosolic N-terminal domain of the Vph1 isoform (Vph1NT) interacts with fragments from the center of Rav1 (amino acids 679–890), both in two-hybrid assays and in pull-downs of expressed fragments (Smardon et al., 2015). In contrast, Stv1NT exhibits little or no interaction with these fragments. This isoform-specificity is functionally important as well. Overexpression of *STV1* results in localization of Stv1-containing V-ATPases to the vacuole (Manolson et al., 1994). When *STV1* was overexpressed in a *rav1*Δ mutant, Stv1-containing V-ATPases, unlike Vph1-containing V-ATPases, were assembled and active in the vacuole, suggesting that assembly of Stv1-containing V-ATPases is not dependent on RAVE (Smardon et al., 2014). Significantly, Stv1-containing V-ATPases also show relatively little disassembly in response to glucose deprivation (Kawasaki-Nishi et al., 2001), which may account for their RAVE independence. The isoform-specificity of the RAVE complex may help to explain the “partial” Vma^–^ phenotype of the *rav1*Δ and *rav2*Δ mutations. The specificity is important because targeting distinct V-ATPase subpopulations has been a long-term therapeutic goal, and these data indicate that RAVE regulates a specific subpopulation of V-ATPases. Higher eukaryotic Rabconnectin-3 complexes might target specific isoforms and subpopulations as well.

Taken together, these experiments indicate that the yeast RAVE complex interacts with all three parts of the V-ATPase that are separated during reversible disassembly: the V_1_ complex, subunit C, and membrane bound V_o_ complexes containing Vph1. These interactions provide a framework for understanding RAVE function, but by themselves cannot indicate how RAVE might promote V-ATPase assembly.

### How Does the RAVE Complex Catalyze V-ATPase Assembly?

V-ATPase reassembly requires signal-dependent restoration of interactions between the V_1_ complex, subunit C, and membrane-bound V_o_, and defining how and where the RAVE complex intervenes in this process is complex. As described above, the RAVE and V_1_ complexes co-precipitate from cytosolic fractions. As expected, there is more V_1_ in complex with RAVE in the cytosol of glucose-deprived cells than in glucose-replete cells, since V_1_ is partially released from the membrane upon glucose deprivation (Smardon et al., 2002). However, the interaction between the two complexes is not intrinsically glucose sensitive, because in a mutant where V_1_ is always cytosolic, there is no difference in RAVE-V_1_ interaction in the presence and absence of glucose (Smardon et al., 2002). In contrast, the interaction of RAVE with Vph1-containing V_o_ complexes at the vacuolar membrane is glucose-sensitive (Smardon et al., 2015). GFP-tagged Rav1 and Rav2 subunits are cytosolic in glucose-deprived cells but are recruited to the vacuolar membrane when glucose is added back to cells. Importantly, glucose-dependent localization of RAVE to the membrane occurs even in subunit E and G mutants that prevent the RAVE-V_1_ interaction or in subunit C mutants (Smardon et al., 2015; Jaskolka and Kane, 2020). These results indicate that the interaction between the RAVE complex and V_o_ is glucose-sensitive and suggest that glucose signaling targets this interaction to promote reassembly. It was previously shown that glucose itself is not a signal, since bypassing the initial step of the glycolytic pathway still permits reassembly. Interestingly, glycolytic flux may be critical for signaling reassembly, as indicated by mutations in one of the two phosphofructokinase subunits (Pfk2). In a *pfk2*Δ mutant, reassembly upon glucose readdition is incomplete, and RAVE-V_1_ complexes accumulate in the cytosol (Chan and Parra, 2014). However, higher glucose concentrations, which restore glycolytic flux, can restore V-ATPase activity in the *pfk2*Δ mutant (Chan et al., 2016). A conserved six amino acid sequence (amino acids 757–762) in the region of Rav1 that binds Vph1NT proved to be critical for targeting RAVE to the vacuolar membrane. However, this sequence appears to be directly involved in binding rather than glucose sensitivity, since a bacterially expressed fragment lacking the sequence also fails to bind Vph1NT *in vitro* (Jaskolka and Kane, 2020). These data provide important clues about the role of RAVE in reassembly, but do not clarify the order of events in RAVE-mediated V-ATPase reassembly.

**FIGURE 4:**
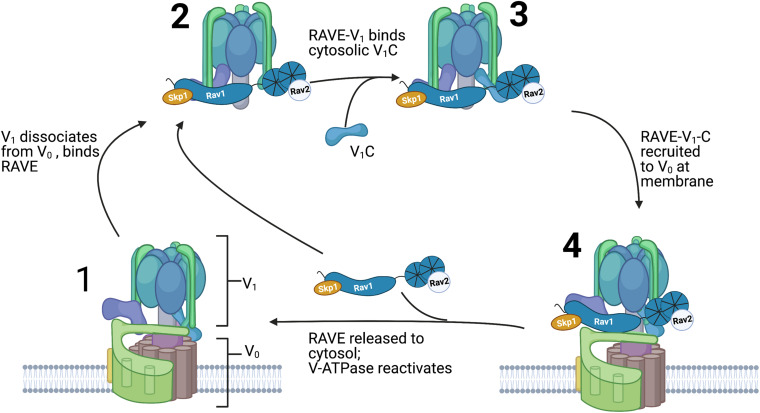
Order of events in RAVE-driven catalysis of V-ATPase reassembly. Assembled and active V-ATPases (1) are dissociated upon glucose deprivation as described in Figure 2. The RAVE complex binds to cytosolic V_1_ complexes (2) and this RAVE-V_1_ complex then binds to subunit C (3). After glucose readdition, RAVE is recruited to V_o_ complexes at the vacuolar membrane and catalyzes recruitment and functional reassembly of V_1_ and subunit C with V_o_ complexes (4). RAVE is then released and able to catalyze another cycle of reassembly.

It is clear from global quantitation of the yeast proteome that Rav1 and Rav2 are present at no more than 10% the level of the V-ATPase (Breker et al., 2013). Thus, RAVE cannot bind stoichiometrically with the V-ATPase but instead must act catalytically. In addition, the limited amount of Rav1 and Rav2 made biochemical studies of the full RAVE complex difficult until a yeast strain capable of inducible overexpression of *RAV1* and *RAV2* was developed (Jaskolka et al., 2021). From this overexpressing strain, it was possible to purify both RAVE alone and RAVE in complex with V_1_. Surprisingly, subunit C did not co-purify with either complex. However, *in vitro* binding experiments demonstrate that RAVE-V_1_ complexes can bind to exogenously supplied subunit C, but RAVE alone binds much less tightly. Taken together, these data suggest that RAVE-V_1_ is an initial intermediate in V-ATPase reassembly, and subunit C subsequently binds to the RAVE-V_1_ complex on the pathway to reassembly. Purified RAVE was able to significantly accelerate functional reassembly of V_1_ complex, subunit C, and V_o_ complexes reconstituted into nanodiscs *in vitro* (Sharma et al., 2019; Jaskolka et al., 2021), but only when the V_1_ complex contained a mutation that prevented subunit H from assuming its “locked” conformation in V_1_. A proposed sequence of events in RAVE-mediated V-ATPase reassembly is shown in Figure 4.

## Rabconnectin-3 Complexes of Higher Eukaryotes

### Genetic Identification of Rabconnectin-3 Subunits

Current data suggests that the Rabconnectin-3 complex is a heterodimer consisting of Rabconnectin-3α and Rabconnectin-3β subunits (Nagano et al., 2002; Kawabe et al., 2003). Early knowledge surrounding Rabconnectin-3 suggested little to no connection to V-ATPase regulation. The first Rabconnectin-3α homolog was identified in *Drosophila melanogaster* (Kraemer et al., 1998). The gene encodes an unusually large protein with a corresponding cDNA of approximately 11.5 kb. It resides on the X chromosome; as a result, it was named DmX (***D****rosophila* ***m****elanogaster* **X** chromosomal gene). Despite its location on a sex determining chromosome, it does not appear that DmX expression is exclusive to any specific developmental stage or sex (Kraemer et al., 1998). The DmX protein is approximately 3400 amino acids with a molecular mass of 380 kDa. A defining characteristic of the DmX protein is the large number of WD40 repeats. WD40 repeats fold to form structures called β-propellers which are composed of several ‘blades’ of antiparallel β-sheets (Xu and Min, 2011). WD40 repeats and β-propellers frequently facilitate protein-protein interactions. The predicted presence of β-propellers suggested that the DmX protein might serve a regulatory or protein-protein scaffolding function.

Sequence analysis suggests that there are several different DmX-‘like’ proteins in other organisms. This includes two in humans of roughly similar molecular weights, and one in yeast which is about half the size. The human proteins were named DMXL1 and DMXL2 (Kraemer et al., 2000), and the yeast protein was later identified as Rav1 (Seol et al., 2001). The most highly conserved region of Rav1 is between amino acids 840 and 1125, and homologous sequences are detected in most eukaryotes. The six amino acid motif in Rav1 that was implicated in binding to Vph1 is present in almost all Rabconnectin-3α homologues (Jaskolka and Kane, 2020), where it is found in a poorly conserved region 88-208 amino acids upstream of the most highly conserved region. Direct sequence homology in the N-terminal β-sheet enriched region is also detectable across multiple organisms. Like humans, mice, zebrafish, and several other higher eukaryotes have two Rabconnectin-3α subunit isoforms called DMXL1 and DMXL2. Other organisms, like *Drosophila melanogaster*, have only a single Rabconnectin-3α isoform. The expression of human DMXL isoforms is widespread, but also displays some tissue enrichment. DMXL2 expression is highly enriched in brain, although it is also expressed in other tissues. DMXL1 is widely expressed, but is enriched in kidney, prostate, and thyroid (Fagerberg et al., 2014).

The Rabconnectin-3β subunit is called WDR7 in humans and is ubiquitously expressed, with highest expression in brain, prostate, and thyroid (Fagerberg et al., 2014). WDR7 is a smaller protein than DMXL1 and 2, with a molecular weight of approximately 164 kDa, in contrast to DMXL2’s 339 kDa. As described in more detail below, it is likely that WDR7 interacts with both DMXL1 and DMXL2. The result is that there is not a single human Rabconnectin-3 complex, but instead two complexes containing different DXML isoforms that may function in different locations or have compartment-specific functions. This is comparable to V-ATPases, which are referred to by a single name but are in fact a heterogenous population containing multiple subunit isoforms. Individual Rabconnectin-3 complexes may contain different combinations of isoforms depending on cell type. For example, Rabconnectin-3 in brain may consist primarily of DMXL2/WDR7 complexes while Rabconnectin-3 complexes in other tissues may be enriched in DMXL1/WDR7. Rabconnectin-3 complexes containing different DMXL isoforms may have different properties and select different V-ATPase subpopulations.

### Discovery of Rabconnectin-3 Complex Functions

The first functional information surrounding mammalian Rabconnectin-3 was discovered several years after the genomic identification of two DMXL proteins in humans (Kraemer et al., 2000). DMXL2 was immunoprecipitated with the Rab3A GAP and GEF (GTPase Activating Protein and Guanine Exchange Factor, respectively) from a rat brain crude synaptic vesicle fraction (Nagano et al., 2002). Rab3A is a small G protein involved in the calcium sensitive release of synaptic vesicle contents from neurons upon stimulation (Geppert et al., 1997), and the connection to Rab3 accessory proteins is the reason for the name Rabconnectin-3. Interaction of DMXL2 with the Rab3 GEF and GAP suggested that the complex plays a role at the synapse, specifically with synaptic vesicles. Several other proteins co-precipitated with DMXL2 including WDR7 (Kawabe et al., 2003). DMXL2 (Rabconnectin-3α) and WDR7 (Rabconnectin-3β) together comprise a synaptic Rabconnectin-3 complex in an apparent 1:1 ratio (Kawabe et al., 2003).

Since the initial identification of Rabconnectin-3, several connections to V-ATPase activity have been discovered while functional links to Rab3 have become less prominent. In a search for mutations affecting tissue organization in Drosophila, Yan et al. (2009) identified loss of function mutations in both Rabconnectin-3α and β. They showed that the observed morphological defects arose from defective Notch signaling, described below, associated with defective endosomal trafficking. Endosomal acidification was affected in the mutants, and the Rabconnectin-3 mutant phenotypes could be phenocopied by a mutation in a V-ATPase subunit. Rabconnectin-3 subunits also co-precipitated with the V-ATPase from fly ovaries (Yan et al., 2009). These results indicated that Rabconnectin-3 complexes can interact with the V-ATPase and regulate organelle acidification in higher eukaryotes, as the RAVE complex does in yeast.

As mentioned earlier, there are likely to be multiple Rabconnectin-3 complexes that contain different DMXL isoforms and vary by tissue. DMXL2 immunoprecipitated V_1_ subunit C, along with several other V_1_ subunits and WDR7, from mouse brains (Li et al., 2012). Both DMXL1 and DMXL2 proteins are present in mouse kidney, although their cellular localization appears to be somewhat different (Merkulova et al., 2015). DMXL1, DMXL2, and WDR7 all co-immunoprecipitated with the V-ATPase from mouse kidney lysates. However, silencing DMXL1 or WDR7 significantly impaired recovery of acidification in cellular organelles after transient treatment with the V-ATPase inhibitor bafilomycin, while DMXL2 silencing had only a modest effect (Merkulova et al., 2015). These data indicate that DMXL1-containing Rabconnectin-3 complexes are important for V-ATPase-dependent organelle acidification (Merkulova et al., 2015) and suggest that DMXL1-containing Rabconnectin-3 complexes facilitate V-ATPase assembly in non-neuronal cell types. Hair cells are specialized epithelial cells that convert physical stimuli into electrical signals. They relay stimuli to the nervous system through synapses with neurons (Hudspeth, 1985). In zebrafish, DMXL2-containing Rabconnectin-3 complexes promote assembly and activity of V-ATPases on hair cell synaptic vesicles (Einhorn et al., 2012). Proper loading of neurotransmitter and the subsequent release of synaptic vesicles require a relatively large concentration of V-ATPases at the synapse that likely require DMXL2 for assembly (Bodzeta et al., 2017). Taken together, these data support the idea that Rabconnectin-3 complexes containing different DMXL isoforms can influence V-ATPase activity in a tissue-specific manner. One possibility is that the ubiquitously expressed DMXL1 more broadly facilitates V-ATPase reassembly, in endosomal membranes, for example. DMXL2, on the other hand, facilitates V-ATPase reassembly under more specialized conditions, such as the presynaptic membranes of hair cells and neurons where the demands on V-ATPases are high.

### Sequence and Structural Similarities and Differences Between Rabconnectin-3 and Yeast RAVE

Based on experimental data, yeast RAVE and higher eukaryotic Rabconnectin-3 complexes share functional similarities. In the absence of structural data for either RAVE or Rabconnectin-3, homology modeling has helped evaluate structural similarities. Based on Phyre2 predictive structural modeling (Kelley et al., 2015), Rav1 and both Rabconnectin-3α and Rabconnectin-3β have common secondary structure features, including a β-sheet-rich N-terminal region followed by a region rich in α-helices which was modeled as an α-solenoid in Rav1 (Figure 3). The N-terminal region of all three proteins can be modeled as a double β-propeller which is attributable to the WD40 repeats present in all three genes. However, unlike Rav1, the larger Rabconnectin-3α and β are also predicted to have WD40 repeats near their C-terminus. As described above, Rabconnectin-3α and Rav1 share three regions of conserved sequence. The most conserved region is in the middle of each protein (corresponding to amino acids 840–1150 of Rav1); this is the region of Rav1 involved in binding V_1_ subunits (Figure 3) and it might play a similar role in Rabconnectin-3α. The Rav1 motif implicated in binding Vph1NT is conserved in Rabconnectin-3α as well (Jaskolka and Kane, 2020). Finally, there is sequence homology between Rabconnectin-3α and Rav1 in the region that binds to Rav2 in RAVE (Smardon et al., 2015; Figure 3). Rabconnectin-3β shares very little direct sequence homology with Rav1, but notably, human WDR7 and yeast Rav1 can be modeled at high confidence to the same target proteins. Although Rabconnectin-3β lacks the region of direct sequence homology with Rav1, it is necessary to facilitate V-ATPase reassembly suggesting that it is not redundant with Rabconnectin-3α (Sethi et al., 2010). It is interesting that Rabconnectin-3 complexes contain two subunits that loosely resemble Rav1.

### Are There Additional Rabconnectin-3 Subunits?

A noticeable difference between Rabconnectin-3 and yeast RAVE is the apparent loss of the Rav2 subunit. As described above, yeast *rav2*Δ mutant cells display a Rav^–^ phenotype similar to that of *rav1*Δ cells (Seol et al., 2001; Smardon et al., 2002). Given the apparent functional importance of Rav2, it is surprising that neither of the Rabconnectin-3 subunits seem to resemble Rav2. However, despite limited direct sequence homology, almost the entire sequence of yeast Rav2 can be modeled with high confidence onto a recent crystal structure of the human Rogdi protein (Lee et al., 2017). Homozygous loss of function mutations in the Rogdi protein are associated with Kohlschutter-Tonz syndrome (Schossig et al., 2012a, b), which is characterized by early onset epilepsy, developmental delay, and defective tooth enamel development (amelogenesis imperfecta). There had been no previous association of Rogdi with the V-ATPase or Rabconnectin-3 complexes, but the strong structural similarities suggest that a potential connection with Rabconnectin-3 should be investigated. Notably, Rogdi, along with DMXL1, DMXL2, and WDR7, was also immunoprecipitated from murine kidney with a V-ATPase subunit antibody, although at a somewhat lower level (Merkulova et al., 2015).

Mammals also encode a homologue of WDR7, WDR72. Human WDR72 (1102 amino acids) is shorter than WDR7, but is 37% identical and 58% similar to WDR7 over the initial 917 amino acids. WDR72 shows strong tissue-specific expression, with high levels of expression in kidney and thyroid (Fagerberg et al., 2014). At present, there is no evidence that WDR72 interacts with Rabconnectin-3 subunits, but there is evidence of involvement in acidification. Mutations in WDR72 are associated with a syndrome characterized by amelogenesis imperfecta and distal renal tubular acidosis (DRTA) (Zhang et al., 2019; Jobst-Schwan et al., 2020). Interestingly, mutations in V-ATPase subunit isoforms that localize to the plasma membrane in the distal renal tubule are also associated with DRTA (Karet, 2002; Jobst-Schwan et al., 2020). Amelogenesis imperfecta may also arise from organelle acidification defects in the ameloblasts that compromise degradation of enamel proteins (Wang et al., 2015). Given the similarity to WDR7 and links to pH control, it is important to investigate the possibility that WDR72 may participate in Rabconnectin-3 complexes, at least in some specific locations.

Although Skp1 is a highly conserved protein and is well-established as a subunit of yeast RAVE, there is little evidence that Skp1 binds to Rabconnectin-3α or β and no clear association between mammalian Skp1 and organelle acidification. The very large size of the mammalian Rabconnectin-3 subunits could make it difficult to visualize a small protein like Skp1 (<30 kDa) on the same SDS-PAGE gel (Kawabe et al., 2003), so it could have been missed. However, Skp1 appears to play a rather peripheral role in yeast RAVE, so it is possible that it is either not required for Rabconnectin-3 activities or its function is replaced by other portions of the larger Rabconnectin-3 subunits.

### Rabconnectin-3 Complexes in Endosomal Signaling

V-ATPase dependent endosomal acidification is essential for cellular homeostasis and developmental processes (Eaton et al., 2021). Two important developmental processes which depend on V-ATPase activity are Wnt and Notch signaling (Yan et al., 2009; Cruciat et al., 2010). The Wnt ligand binds to a receptor complex on the plasma membrane composed of Frizzled and LPR6. Upon binding of the Wnt ligand, activated receptors cluster together at or adjacent to the plasma membrane and recruit downstream proteins into complexes known as signalsomes (Bilic et al., 2007). Receptor clustering requires the phosphorylation of the cytoplasmic tail of LRP6 which then interacts with the protein Dishevelled on the cytosolic side of the membrane (Bilic et al., 2007). Both V-ATPase subunits and V-ATPase-driven endosomal acidification are required for Wnt signal transduction (Cruciat et al., 2010; Stransky et al., 2016). Following Wnt binding to Frizzled and LPR6, the Wnt ligand-receptor complex is endocytosed, and the newly formed vesicle is acidified by V-ATPases. Exposure of the extracellular domain of LPR6 to the acidic environment appears to promote a conformational change that then allows for LPR6 phosphorylation. Vesicle acidification thus enables LPR6 phosphorylation, clustering/signalosome formation and the transduction of the signal relayed by the Wnt ligand. Knockdown of V-ATPase subunits or treatment with the V-ATPase inhibitor bafilomycin inhibited LPR6 phosphorylation and downstream signaling events (Cruciat et al., 2010). Taken together, these data indicate that V-ATPase-mediated organelle acidification is essential for Wnt signaling.

Consistent with its role as regulator of V-ATPase activity, loss of DMXL2 function interferes with Wnt signaling in zebrafish neural crest cells (Tuttle et al., 2014). Paradoxically, in the absence of DMXL2 there are abnormally large but still acidified early endosomal compartments. The effects on Wnt signaling are complex. Relative to the control strain, expression of Wnt target genes initially decreased, but later increased over time (Tuttle et al., 2014). The accumulation of early endosomes may result from a failure of endosomal maturation to lysosomes, resulting in decreased protein turnover. A longer lifetime for activated signalosomes accumulating in the large endosomes could account for the eventual increase in Wnt target gene expression. However, the initial decrease in expression is still unexplained. Importantly, the absence of V_o_ a1 subunit in neural crest cells phenocopies the loss of DMXL2 (Tuttle et al., 2014). This confirms that the loss of DMXL2 exerts its regulatory effects through the V-ATPase, though the precise mechanisms are unknown.

Notch signaling also depends on V-ATPase activity. Following binding of the Notch ligand to the Notch receptor, the extracellular domain is removed, and the transmembrane and intracellular domain is endocytosed. Following endocytosis, the intracellular domain is freed from the membrane by γ-secretase mediated proteolytic cleavage early endosomes (S3 cleavage). After S3 cleavage, the cytoplasmic domain moves to the nucleus and activates transcription of target genes (Kopan, 2012). V-ATPase activity is required for correct Notch signaling but the precise mechanism is somewhat unclear (Yan et al., 2009; Sethi et al., 2010; Vaccari et al., 2010; Wissel et al., 2018). In the absence of functional V-ATPase and Rabconnectin-3, Notch accumulates in late endosomal compartments, leading to a decrease in Notch target gene expression. Comparison with mutants that have a similar phenotype suggests that the endosomal accumulation is not the cause of decreased Notch-dependent transcription (Yan et al., 2009). Instead, Sethi et al. (2010) found that when endosomes are not acidified γ-secretase is unable to cleave Notch at S3. This prevents Notch from activating its transcriptional program and leads to the accumulation of unprocessed Notch in late endosomes. Loss of *Drosophila* Rabconnectin-3β phenocopies mutations in core V-ATPase subunits, as well as Rabconnectin-3α (Yan et al., 2009; Sethi et al., 2010). This phenotype demonstrates that loss of a fully functional Rabconnectin-3 complex leads to a decrease in V-ATPase activity and a concomitant increase in endosomal pH that compromises Notch signaling.

Endosomal acidification is also exploited by viruses to drive conformational changes that allow release of their genetic material into the cell. V-ATPase activity can be manipulated by viruses to promote endosomal acidification and viral entry (Marjuki et al., 2011). V-ATPase assembly levels have been associated with levels of viral infection. At high glucose levels, V-ATPase assembly and cellular infection by influenza virus increased, while reduced V-ATPase assembly in the presence of glycolytic inhibitors decreased viral infection (Kohio and Adamson, 2013). In line with these results, a recent genome-wide CRISPR screen to identify host factors necessary for influenza infection found that a CRISPR knockout of WDR7 compromised influenza entry to a similar extent as knockouts of V-ATPase subunits, and also decreased V-ATPase assembly (Li et al., 2020). This result suggests that decreasing the activity of Rabconnectin-3 could have antiviral effects.

Finally, in addition to V-ATPases, it has been found that Rabconnectin-3 interacts with proteins responsible for calcium signaling and calcium sensitive exocytosis. Both Rabconnectin-3 subunits interact with and appear to modulate the activity of CAV2.2, a transmembrane calcium channel (Gandini et al., 2019). Another protein, CAPS1, interacts with WDR7 and loss of CAPS1 impairs dense core vesicle acidification (Crummy et al., 2019). These interactions suggest that Rabconnectin-3 may serve other functions beyond facilitating V-ATPase reassembly, potentially explaining some of the structural differences compared to yeast Rav1.

### Rabconnectin-3 and Disease

So far, pathologic mutations in the Rabconnectin-3 complexes are primarily in DMXL2. Mutations in DMXL2 have been connected to Ohtahara Syndrome, nonsyndromic hearing loss, and neuroendocrine dysfunction (Tata et al., 2014; Chen et al., 2017; Esposito et al., 2019). Ohtahara syndrome, also known as early infantile epileptic encephalopathy, is an epileptic syndrome which presents very early in life (Beal et al., 2012). V-ATPases are critical for the loading and maturation of synaptic vesicles and Rabconnectin-3α is important for assembly of synaptic vesicle V-ATPases, which could suggest that loss of synaptic vesicle function is the basis of the aberrant neurological activity associated with DMXL2 deficiency. In addition, silencing of DMXL2 in mouse hippocampal neurons resulted in impaired autophagy and defective lysosomes (Esposito et al., 2019), both of which could be associated with neurodegeneration. Non-syndromic hearing loss has also been observed in individuals with DMXL2 mutations (Chen et al., 2017). Mutations in the V_o_ a4 and V_1_ B1 V-ATPase subunit isoforms have previously been associated with hearing loss, suggesting that V-ATPase activity is required for the maintenance of correct endolymph pH (Karet et al., 1999; Stover et al., 2002; Vargas-Poussou et al., 2006). It is not certain whether hearing loss caused by DMXL2 mutations is the result of deviations in endolymph pH or defects in synaptic vesicle acidification, akin to what was observed in zebrafish hair cells (Einhorn et al., 2012). Mutations in DMXL2 have also been associated with delayed puberty, decreased fertility and multiple neuroendocrine deficiencies, including low insulin and reduced release of gandotropin-releasing hormone (Tata et al., 2014). These defects could arise from decreased V-ATPase activity in specific locations but could also flow from connections with Rab3 and its role in regulated exocytosis. Copy number variations in DMXL1 have been associated with glaucoma (Davis et al., 2011). DMXL1 is expressed in multiple parts of the eye, and the mechanism of its possible involvement in glaucoma has not been assessed.

Overexpression of DMXL2 has been observed in breast cancer patients that are resistant to endocrine therapy (Faronato et al., 2015). In this context, DMXL2 appears to drive Notch hyper-activation and promote acquisition of epithelial to mesenchymal transition phenotypes. Significantly, downregulation of either DMXL2 or V-ATPase activity reduces upregulation of Notch targets and invasion phenotypes in breast cancer cell lines (Faronato et al., 2015).

## Future Directions and Prospects for RAVE/Rabconnectin-3 Research

There are certainly many questions about the structure, mechanism, and physiological roles of the RAVE/Rabconnectin-3 complexes to be answered. Work on yeast RAVE has provided a number of insights into RAVE interactions with the V-ATPase and its role in V-ATPase reassembly. However, important questions such as how RAVE orchestrates the assembly of the disassembled V-ATPase pieces and how glucose signals V-ATPase reassembly remain unanswered. High resolution structural information would help address how RAVE binds its partners and catalyzes reassembly. Yeast RAVE may be the best structural target because it is a smaller complex with a defined subunit composition that can be overexpressed and purified (Jaskolka et al., 2021).

Although yeast RAVE provides a paradigm for some aspects of the function of higher eukaryotic Rabconnectin-3 complexes, there are key issues that still must be addressed directly on Rabconnectin-3. First, a better understanding of the subunit composition of Rabconnectin-3 complexes in different tissues is critical, and it is still not clear that the full set of subunits and isoforms have been identified. In addition, despite areas of sequence and structural homology, the Rabconnectin-3 complexes are much larger than yeast RAVE. It is not clear whether there is a “RAVE core” dedicated to V-ATPase interactions within Rabconnectin-3 or whether these larger subunits endow the Rabconnectin-3 complexes with other functions. It will be very interesting to determine whether Rabconnectin-3 complexes distinguish between V_o_ a-subunit isoforms as yeast RAVE does. If they do, then manipulating Rabconnectin-3 interactions with the V-ATPase might provide a means of targeting the activity of specific V-ATPase subpopulations, an important step toward therapeutic targeting of V-ATPase function in specific locations. Integrating the diverse signals implicated in governing V-ATPase reversible disassembly with Rabconnectin-3 interactions in mammalian cells is also important. The physiological range of Rabconnectin-3 function has not been fully addressed, but the association of subunit mutations with disease will motivate this research. Mice with a homozygous deletion of DMXL2 failed to feed and died shortly after birth (Gobe et al., 2019), but tissue-specific targeted knockouts have been, and will continue to be, informative (Esposito et al., 2019; Gobe et al., 2019). There is still much to be learned about the RAVE and Rabconnectin-3 complexes, but the results so far highlight their essential role in multiple physiological processes and their promise as therapeutic targets.

## Author Contributions

SW, PK, and MJ prepared the figures and wrote and edited the manuscript. All authors contributed to the article and approved the submitted version.

## Conflict of Interest

The authors declare that the research was conducted in the absence of any commercial or financial relationships that could be construed as a potential conflict of interest.
